# New Insights into Thermodynamics of Solutes in Neat and Complex Solvents

**DOI:** 10.3390/molecules27186131

**Published:** 2022-09-19

**Authors:** Piotr Cysewski, Tomasz Jeliński, Maciej Przybyłek

**Affiliations:** Department of Physical Chemistry, Pharmacy Faculty, Collegium Medicum of Bydgoszcz, Nicolaus Copernicus University in Toruń, Kurpińskiego 5, 85-950 Bydgoszcz, Poland

Solubility is one of the most important physicochemical properties, both from a practical and theoretical perspective. Various fields benefit from the knowledge of this fundamental property, including pharmaceuticals, food technology, materials engineering and purification or separation techniques, including green extraction. Furthermore, solvent selection is important in sustainable technologies design, which was also addressed in the aims list of this Special Issue. Since the early 1990s, a significant increase in solubility studies can be observed, and the interest in this topic is growing every year (Figure 1). This Special Issue contributes to the field by providing a few important and interesting studies on a variety of systems. In the presented collection, two basic approaches to the thermodynamic description of solubility can be distinguished: the “classical” one, related to the application of well-known equations for data fitting, and molecular modeling-based methods. Both approaches are interesting and worth exploring. This editorial, providing an invitation for the reader to dive into the Special Issue, is ordered by the date of publication.

The first paper published in this Special Issue was the work by Ghazwani et al. [1]. This study dealt with the topic of solubility of trans-resveratrol, a compound with many different therapeutic activities in several neat solvents including water, as well as propylene glycol mixtures with water. Additionally, the thermodynamic properties of trans-resveratrol dissolution were studied, and the experimental solubility values were correlated with different solubility models. Neat propylene glycol was found to be the most effective solvent for trans-resveratrol dissolution, and the dissolution process was found to be endothermic and entropy-driven.

In a similar work by the Ghazwani et al. research group [2], the solubilizing behavior of thymoquinone, a compound with antioxidant, anti-inflammatory, anticancer and other important biological activities was examined in various binary combinations of isopropanol and water. Additionally, the Hansen solubility parameters and thermodynamic behavior of the above compound were determined in the utilized binary mixtures. The solubility of thymoquinone was found to increase with the increasing content of isopropanol in the mixtures. The experimental solubility was regressed with the use of different solubility models, including van’t Hoff, Apelblat, Yalkowsky–Roseman, Buchowski–Ksiazczak λh, Jouyban–Acree, and Jouyban–Acree–van’t Hoff models. The authors found that the maximum molecular solute–solvent interactions occurred in the case of thymoquinone and isopropanol rather than in water. It was also noted that the dissolution process of thymoquinone was endothermic and entropy-driven in all studied mixtures and neat solvents.

The work by Przybyłek et al. [3] focused on the studies of the thermodynamic properties of phenacetin, analgesic and antipyretic agent, in the solid state, as well as in neat and binary solvents. The authors found that all studied neat solvents offered a substantial solubility increase of phenacetin compared to water. They also concluded that all studied binary solvents experienced strong positive non-ideal deviations from the algebraic rule of mixing and observed a synergistic effect in the case of acetonitrile and 1,4-dioxane, enhancing phenacetin solubility compared to pure solvents. Additionally, a key aspect of the study was the determination of the temperature-related heat capacity values measured for both the solid and melt states of phenacetin by using the temperature-modulated differential scanning calorimetry (TMDSC) technique. This, in turn, allowed for the determination of such parameters as ideal solubility or activity coefficients. The authors discussed the factors which affect the accuracy of the mentioned values in terms of different models of specific heat capacity differences and concluded that different properties have varying sensitivity, affecting the accuracy of heat capacity values. While these considerations are related particularly to phenacetin, they can possibly be extended to other solids.

Nutraceuticals are frequently studied substances, and examples of this subject can also be found in this Special Issue. Furia et al. [4] studied the effect of the ionic strength on the aqueous solubility of popular phenolic acids (caffeic, ferulic, syringic, vanillic gallic, and p-coumaric acid). Based on the measurements, the authors determined Setschenow coefficients. They also showed that the salt solutions used in the study containing Na^+^, Cl^−^ and ClO_4_^−^ can act as effective co-solvents of phenolic acids, which is important from a practical viewpoint.

In the study by Ortiz et al. [5], the solubility of a popular sulfonamide, sulfamethazine, in acetonitrile–methanol binary mixtures was examined at several temperatures. In the study, popular “classic” models based on thermodynamic formalism were also applied. Noteworthily, the quality of fitting was evaluated using the above-mentioned mean relative deviation parameter. According to this analysis, the Wilson models and NRTL method are very suitable for sulfamethazine solubility data interpretation. Additionally, the authors performed DSC measurements of fusion parameters (melting point, enthalpy of fusion). Noteworthily, these parameters were determined for the equilibrated solid residues.

The paper by Rahimpour et al. [6] raised an important and very basic issue, namely solute/solvent composition units in solubility research. In the study, popular units, grams per liter [g/L], mole and molar fraction were used for expressing the solubility values, whereas the solvent concentrations were expressed as volume, mass and molar fraction. The authors analyzed 79 datasets and used the Jouyban–Acree model. The fitting quality was evaluated using several parameters: error in logarithmic scale, error in arithmetic scale, percentage of mean relative deviation, logarithmic scale RMSD and relative mean square deviation of arithmetic scale. As it was established, the mean relative deviation parameter is the most reliable and comprehensive measure. 

The aspect of green solvent screening was considered by Przybyłek et al. by formulating and validating a new screening protocol for green and effective solvent screening [7]. The solubility of benzamide analogs as the exemplary set of bio-active compounds was studied both experimentally and theoretically using the conductor-like screening model for realistic solvents (COSMO-RS) approach. The applied protocol successfully identified 4-formylmopholine and its aqueous binary mixtures as environmentally friendly and effective solvents for the processing of benzamide analogs.

Haq et al. [8] examined the solubility and thermodynamics of febuxostat (FBX) in a variety of mono solvents at 298.2–318.2 K. The obtained data were interpreted in terms of such theoretical models as van’t Hoff, Buchowski-Ksiazczak λh, and Apelblat. The procedure for solubility optimization led to overall error values for van’t Hoff, Buchowski-Ksiazczak λh, and Apelblat models. In addition, thermodynamic analysis was performed to obtain detailed characteristics of the nature of dissolution process in the studied solvents. The authors emphasized that PEG-400 was found as the optimal co-solvent for FBX solubility, as it exhibits the strongest solute–solvent interactions.

Finally, the authors of this editorial [9] provided a form of solubility of published solubility data curation by the application of the COSMO-RS-DARE protocol (DARE—dimerization, aggregation, and reaction extension) supported with external experimental validation. The neat solvents’ solubility of coumarin, a naturally occurring lactone-type benzopyrone with various applications in the pharmaceutical, food, perfume, and cosmetics industries, was critically examined. The paper’s findings not only rely on the appropriate selection of more reliable solubility data, but also provide a theoretical framework for consistency inspection. The proposed procedure extends the range of applicability of COSMO-RS-DARE to new areas of application. A linear correlation between model interaction parameters with molecular descriptors and the distance between the solute and solvent in the Hansen solubility space, R_a_, was also highlighted.

We would like to thank all of the authors who contributed to the “New Insights into Thermodynamics of Solutes in Neat and Complex Solvents” Special Issue of *Molecules*, as well as all the reviewers who provided constructive remarks and the staff of *Molecules* at MDPI for their professional assistance. We hope that the articles published in this Special Issue will be of much interest to the scientific community and will contribute to the dissemination of knowledge on this topic among researchers. 

## Figures and Tables

**Figure 1:**
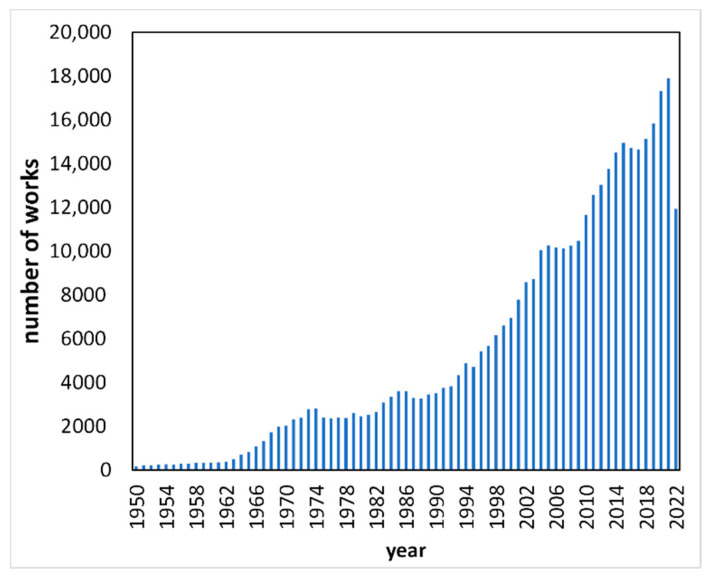
Number of scientific works where the “solubility” term appeared in abstract, title or keywords in 1950–2022 (Scopus, august 2022).

## Data Availability

All data are available in the paper.
